# Pre-Surgery Cortisol Levels as Biomarker of Evolution after Bariatric Surgery: Weight Loss and Weight Regain

**DOI:** 10.3390/jcm13175146

**Published:** 2024-08-30

**Authors:** Anna Casteràs, Enzamaria Fidilio, Marta Comas, Alba Zabalegui, Vanesa Flores, Marina Giralt, Noelia Díaz-Troyano, Roser Ferrer, Ramon Vilallonga, Andreea Ciudin, Betina Biagetti

**Affiliations:** 1 Endocrinology and Nutrition Department, Hospital Universitari Vall d’Hebron, Passeig de la Vall d’Hebron 119-121, 08035 Barcelona, Spain; anna.casteras@vallhebron.cat (A.C.); enzamaria.fidilio@vallhebron.cat (E.F.); marta.comas@vallhebron.cat (M.C.); alba.zabalegui@vallhebron.cat (A.Z.); vanesapaola.flores@vallhebron.cat (V.F.); andreea.ciudin@vallhebron.cat (A.C.); 2 Department of Medicine, Universitat Autònoma de Barcelona, Cerdanyola del Vallès, 08193 Barcelona, Spain; roser.ferrer@vallhebron.cat (R.F.); ramon.vilallonga@vallhebron.cat (R.V.); 3 Diabetes and Metabolism Research Unit, Vall d’Hebron Insitut de Recerca, 08035 Barcelona, Spain; 4 Biochemistry Department, Hospital Universitari Vall d’Hebron, 08035 Barcelona, Spain; marina.giralt@vallhebron.cat (M.G.); noelia.diaz@vallhebron.cat (N.D.-T.); 5 Endocrine, Metabolic and Bariatric Unit, General Surgery Department, Hospital Universitari Vall d’Hebron, 08035 Barcelona, Spain

**Keywords:** dexamethasone suppression test, DST 1 mg, morning serum cortisol, bariatric surgery, weight regain, weight loss, severe obesity

## Abstract

**Background:** Bariatric surgery (BS) is effective for achieving significant weight loss. However, weight regain (WR) is an emerging problem. **Objective:** To assess the prognostic value of morning serum cortisol, a 1 mg dexamethasone suppression test (DST), 24 h urinary free cortisol (UFC) and late-night salivary cortisol (LNSC) in a cohort of patients with severe obesity (pwSO) undergoing BS in terms of weight loss and WR. **Methods:** Patients scheduled for BS underwent the following procedures at baseline, 12 months and 24 months after BS: medical history, anthropometric data, blood analysis and cortisol tests. We evaluated total weight loss (TWL) ≥ 30% at 1 year and WR after 2 years as an increase of ≥10% of the maximum weight lost. **Results**: In total, 142 subjects were included; 101 (71.1%) were females and the mean age was 45.9 ± 9.2 years. Up to 76.8% of subjects achieved ≥30% TWL, without statistically significant differences in DST results or morning serum cortisol, UFC or LNSC levels. However, a higher pre-surgery morning serum cortisol level was a significant predictor of a WR ≥ 10% (cortisol 17.8 [IQR 13.1–18.5] vs. 12.0 [IQR 8.8–15.8] μg/dL; *p* < 0.01); OR of 1.216 (95% CI 1.069–1.384); AUC [0.761, CI: (0.616–0.906); *p* < 0.01]. A cut-off value of cortisol > 13.0 μg/dL was predictive of a WR ≥ 10% (sensitivity 0.71; specificity 0.63). **Conclusions:** No cortisol test was useful in predicting weight loss; however, the pre-surgery morning serum cortisol level was able to predict a WR ≥ 10% in a cohort of pwSO 2 years after BS. A cut-off value of cortisol > 13 μg/dL might be an easy tool to identify patients at higher risk of WR, enabling healthcare providers to implement tailored, long-term strategies to minimize this outcome.

## 1. Introduction

Obesity is a complex, multifactorial, chronic relapsing disease whose prevalence is continuing to increase in an alarming manner worldwide. In Spain, almost one fourth of the population fits the definition of obesity, with a body mass index (BMI) > 30 kg/m^2^, and severe obesity (BMI > 40 kg/m^2^) has a prevalence of 1.2% among adults [1,2]. Bariatric surgery induces significant weight loss, on average 20–40% of total body weight, in a short period of time and may improve or resolve associated comorbidities as well as reduce mortality [3,4,5]. In the last decades, BS performance increased worldwide. However, a significant proportion of patients (15–35%) do not achieve their weight loss goals following the surgery [6]. The factors contributing to this lack of success are multifaceted and include age, gender, genetics [7], comorbidity rate, psychological profile, lifestyle and socioeconomic status [8,9,10,11]. In addition, in the long term, weight regain (WR) becomes a frequent problem, the true prevalence remains unknown as the data vary on how to calculate and define relevant WR [12,13,14]. It has been reported that five years after reaching nadir weight, more than half of cases regain over 20% of the maximum weight lost or more than 15% of the nadir weight [15]. There are several factors that can contribute to WR such as worse adherence to diet and exercise plans, pathological patterns of food intake, younger age and psychological factors like anxiety, depression and addictive behaviors, as well as surgical factors. Despite the growing number of published predictive models for weight loss after BS, there is still a lack of comprehensive scientific consensus on the effectiveness of these tools to select the best candidates [16].

Recently increased hypothalamic–pituitary–adrenal (HPA) tone has been associated with poor outcomes after BS [17,18]. In healthy individuals, glucocorticoid synthesis and secretion follow a circadian rhythm, with the highest levels in the morning and the nadir at around midnight. Obesity has been closely associated with dysregulation of the HPA axis, the neuroendocrine system that controls the body’s stress response. On the other hand, obesity may influence cortisol metabolism, due to a higher distribution volume, greater cortisol clearance and increased tissue-specific cortisol activation through 11β-hydroxysteroid dehydrogenase enzyme [19,20,21,22]. This hormonal imbalance can contribute to the development and maintenance of obesity through several mechanisms. Excessive cortisol promotes the accumulation of visceral fat, increases insulin resistance and stimulates appetite, particularly for calorie-dense, palatable foods [23]. Additionally, dysregulation of the HPA axis can impair energy expenditure, disrupt circadian rhythms and contribute to emotional eating behaviors, further exacerbating weight gain, generating a vicious cycle [24].

There are several ways to assess HPA axis; the most used are morning serum cortisol, dexamethasone suppression test (DST), 24 h urinary free cortisol (UFC) and late-night salivary cortisol (LNSC). Serum cortisol is highly variable according to the circadian rhythm; it is also affected by factors such as stress, diet and exercise and has a bidirectional link to medical conditions such as type 2 diabetes mellitus (T2D) and obstructive sleep apnea syndrome (OSAS). Several studies have observed lower morning cortisol levels in patients with severe obesity (pwSO); however, it remains unclear whether this is an adaptative phenomenon [25,26]. DST with overnight administration of 1 mg dexamethasone (23:00 h) is considered one of the diagnostic gold standard tests of autonomous cortisol secretion and is frequently used in clinical practice [27,28]. While obesity’s impact on the DST response has raised questions about the appropriate dexamethasone dosing [29,30,31,32], many studies report that the performance of 1 mg DST in pwSO is similar to that in normoweight subjects [33]. UFC behaves in a U shape manner, increasing with higher BMI, mainly due to increased cortisol clearance, leading to pseudohypercortisolism in some cases [34,35]. On the other hand, the salivary cortisol concentration mirrors that of serum free cortisol and is easily obtained. A LNSC at the nadir (23:00) is a sensitive test to assess hypercortisolism [27], but false positive results are found with increasing age, hypertension and T2D [36]; nevertheless, the obesity impact remains unknown.

On the basis that cortisol has been implicated in obesity and metabolic syndrome, we asked whether cortisol levels or rhythm could be behind the results of BS. To our knowledge, there are few studies on the performance and prognostic value of pre-surgery cortisol and HPA status on weight loss following BS, and none have addressed WR.

Taking into account all the above considerations, we hypothesize that cortisol levels could serve as a predictive factor for weight loss and regain in pwSO undergoing BS. Thus, the present study aimed to evaluate if cortisol levels at baseline or after a 1 mg DST, late-night salivary or 24-h UFC (1) can predict weight loss 12 months after BS and (2) can predict WR at 24 months.

## 2. Materials and Methods

Patients: This prospective study included consecutive pwSO scheduled for BS at our site between June 2019 and January 2022. Clinical data were collected prospectively after institutional review board approval. Informed consent was obtained from all subjects involved in the study. BS was carried out by a trained surgeon in the setting of a comprehensive multidisciplinary program. The BS technique was individually chosen by the hospital’s obesity committee, mainly Roux-en-Y gastric bypass (RYGBP) or sleeve gastrectomy (SG).

Inclusion criteria: Subjects were 18 to 65 years old with a BMI > 40 kg/m^2^ or ≥35 kg/m^2^ and with obesity-related comorbidities.

Exclusion criteria: Contraindications for BS were as per protocol, in addition to overt clinical endogenous hypercortisolism, corticosteroid treatment or drugs that alter CYP3A4 (e.g., oral contraceptive (OC) treatment). Patients with known adrenal adenoma or pituitary disease were also excluded from the study. Subjects with depression or psychiatric treatments were also excluded.

Study design and measurements: All patients underwent the following procedures at baseline, 12 months and 24 months after the BS: complete medical history, anthropometric data and blood test analysis. We evaluated, in all patients, after 12 h of fasting, baseline morning serum cortisol levels and after 1 mg DST, as well as LNSC and UFC. The DST was conducted as usual [28]; on the following morning, a cortisol ≤ 1.8 µg/dL would exclude Cushing’s syndrome with high sensitivity but lower specificity [37].

Serum cortisol was measured by chemiluminescence immunoassay (Atellica IM 1600, Siemens Healthineers, Tarrytown, NY, USA), with reference values 5.27–22.45 μg/dL. Late-night salivary cortisol was measured by ELISA (SLV-2930, DRG Instruments GmbH, Marburg, Germany), with normal reference values at 23.00 h < 1 ng/mL, and 24 h UFC was measured by chemiluminescence immunoassay (Liaison XL; DiaSorin, Saluggia, Italy) after extraction with dichloromethane, with reference values 12.8–82.5 µg/24 h.

Definition of variables: Body mass index (BMI) was calculated as weight (kg) divided by the square of height (m). Waist-to-hip ratio (WHR) was also performed. We determined percentage of total weight loss (%TWL) and percentage of excess weight loss (%EWL) at 12 and 24 months after BS. The %TWL was calculated as: (initial weight minus follow-up weight/initial weight) × 100. To calculate the %EWL, we used the following equation: amount of weight loss/excess body weight × 100. Excess body weight (EW) was calculated according to a BMI of 25 kg/m^2^: basal weight-(25*height^2^). WR was determined at 24 months as a percentage of increase of the maximum lost weight at the nadir. An increase of ≥10% was considered clinically relevant [15,38]. Delta cortisol means the subtraction of morning cortisol minus post-DST cortisol. We defined the previous registered diagnosis of T2D as the taking of hypoglycemic agents or a fasting plasma glucose concentration ≥ 126 mg/dL or a hemoglobin A1c (HbA1c) level ≥ 6.5% according to ADA recommendations [39]. Hypertension was defined as office systolic blood pressure values ≥ 140 mmHg and/or diastolic blood pressure values ≥ 90 mmHg or already on antihypertensive drugs [40]. OSAS was evaluated by pneumologists and classified as present if apnea-hypopnea index ≥ 15 [41]. Continuous positive airway pressure (CPAP) use was also reported.

Endpoints definition: Optimal weight loss was considered as TWL ≥ 30% and EWL ≥ 75% at 12 months [42,43]. Early WR was determined at 24 months as continuous and binary variable, setting a cut-off increase of ≥10% of the nadir lost weight.

Statistical analysis: Continuous variables were tested for normality using Shapiro–Wilk and expressed as medians and interquartile range [IQR] or means with standard deviations (mean ± SD), as appropriate. Categorical data were described as frequencies (number, %). Comparisons between 2 groups were performed with Student’s *t*-test/Mann–Whitney U test for quantitative variables, and the Pearson’s chi-squared/Fisher exact test for categorical variables as appropriate. The relationship between continuous variables was examined by the Pearson linear correlation test or Spearman’s correlation as needed. Univariate or multivariate logistic regressions were used to establish associations between the cortisol levels and/or clinical variables and the changes of weight and expressed as OR and 95% confidence interval. ROC curves were built to show the performance of the obtained data and determine cut-offs values. A *p* value < 0.05 was considered statistically significant. The data were analyzed using IBM SPSS Statistics (26th version).

## 3. Results

1. Baseline characteristics according to gender.

The study included a total of 142 subjects, consisting of 101 (71.1%) females, with a mean age of 45.9 ± 9.2 years. Baseline characteristics are shown in Table 1. Males were significantly older than females (*p* = 0.04). Although BMI was similar between genders, men had more EW and a higher WHR (<0.01). With respect to comorbidities, men had more hypertension (*p* < 0.01) and used more CPAP (*p* = 0.03) than women, but when adjusting by age, CPAP treatment lost statistical significance. Regarding cortisol levels, morning serum cortisol and LNSC were similar among genders. Interestingly, males exhibited higher cortisol levels in both the DST and UFC compared to females. However, in the linear regression model for the DST, when adjusted by age, BMI, EW, WHR, T2D, hypertension and CPAP use, this significance was not maintained. Similarly, gender lost statistical significance for UFC when adjusted by the factors mentioned above.

2. Preoperative factors predicting weight loss at 12 months after BS

Table 2 compares patients who achieved a TWL ≥ 30% to patients with a TWL of less than 30% at 12 months after bariatric surgery. The majority (76.8%) were able to lose at least 30% of their total weight. There were no significant differences between the two groups in terms of gender distribution, age or the type of BS. Regarding the baseline degree of obesity, subjects with a greater BMI and more EW achieved ≥30% TWL more often. Biochemical parameters including morning serum cortisol, DST, UFC and LNSC were similar between those achieving ≥30% and those achieving <30% TWL. These results remained non-statistically significative after adjusting by BMI and EW.

3. Preoperative factors predicting weight regain at 24 months after BS

Table 3 compares the baseline characteristics of patients who experienced WR of ≥10% of their maximum lost weight 2 years after BS to those who did not. The raw analysis revealed that WR was not associated with gender, age, type of surgery, initial BMI, EW or WHR. Additionally, the presence of T2D, OSAS or hypertension did not significantly influence WR. Furthermore, no significant differences were observed between the two groups in terms of inflammatory markers, DST, LNSC or UFC.

**Figure 1:**
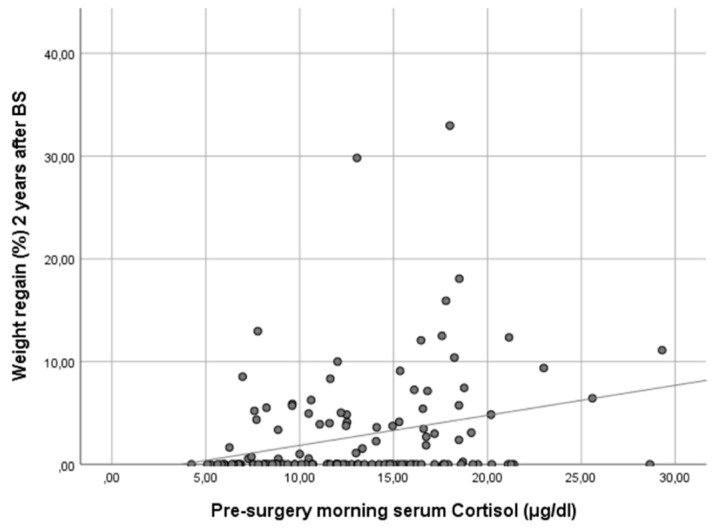
Scatter plot showing the correlation of baseline morning serum cortisol and weight regain at 24 months after bariatric surgery. Figure legend: This scatter plot illustrates the relationship between baseline morning serum cortisol levels and the percentage of maximum lost weight regained at 24 months after bariatric surgery. The data points represent individual patients, and the trend line indicates a positive correlation between the two variables. Spearman’s correlation analysis reveals a coefficient (ρ) of 0.25 (*p* < 0.01), suggesting that higher baseline morning serum cortisol levels are associated with a greater percentage of weight regain 2 years post-surgery.

**Figure 2:**
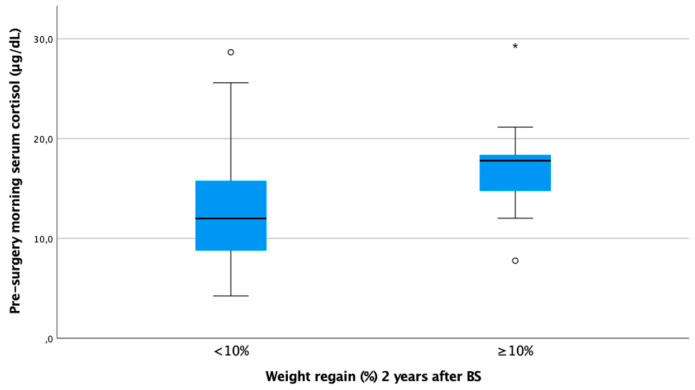
Boxplot of morning serum cortisol according to weight regain ≥ 10% at 24 months after bariatric surgery. Figure legend: Baseline cortisol was statistically significantly higher in subjects with WR ≥ 10% 24 months after BS (17.8 [IQR 13.1–18.5] vs. 12.0 [IQR 8.8–15.8]).

In our cohort, it is noteworthy that only baseline morning serum cortisol was associated with a WR ≥ 10% in both genders in a statistically significant way. Subjects with higher baseline cortisol showed more regain of the lost weight as a continuous variable (ρ 0.25, *p* < 0.01) (Figure 1). In other words, baseline serum cortisol was higher in patients experiencing a WR ≥ 10% (cortisol 17.8 [IQR 13.1–18.5] vs. 12.0 [IQR 8.8–15.8], *p* < 0.01) (Figure 2).

**Figure 3:**
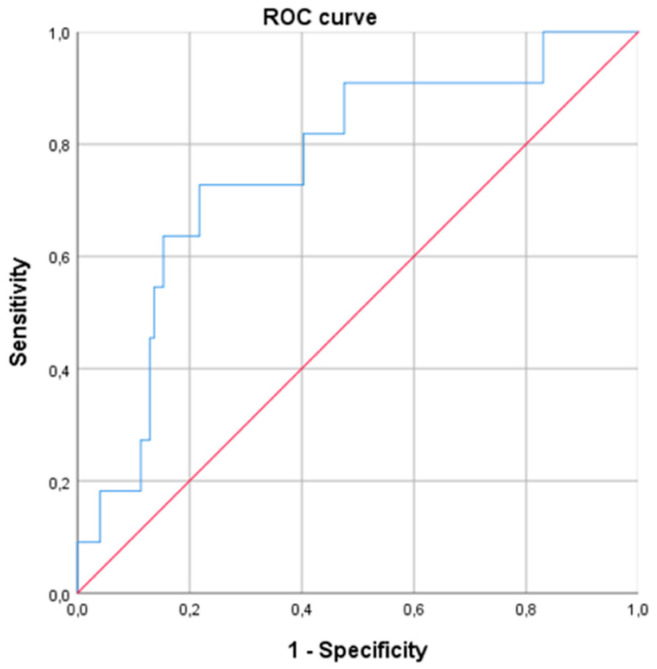
Receiver operating curve (ROC) for prediction of weight regain ≥ 10% at 24 months after bariatric surgery based on baseline morning serum cortisol levels (AUC of 0.761 [0.616–0.906], *p* < 0.01).

In the logistic regression model of WR ≥ 10%, including gender, age, BMI, EW, type of surgery and HOMA, morning serum cortisol remained significant, with an OR of 1.216 (95% CI 1.069–1.384; *p* < 0.01) (Table 4). This means that for every one-unit increase in morning serum cortisol concentration, the probability of experiencing WR increased by 21.6%. The ROC curve using, as a unique variable, the morning serum cortisol level predicted a WR ≥ 10% with an AUC of 0.761 [0.616–0.906], *p* < 0.01 (Figure 3). A cut-off value of cortisol > 13.0 μg/dL was predictive of a WR ≥ 10% (sensitivity 0.71; specificity 0.63; positive predictive value 0.13; negative predictive value 0.96, odds 0.08).

## 4. Discussion

Our study evaluated the relationship of several cortisol studies with the evolution after BS in terms of weight loss at 12 months and WR at 24 months after BS. Neither baseline morning cortisol levels nor cortisol levels after DST, LNSC or UFC predicted the initial 12-month weight loss outcome; however, higher preoperative morning serum cortisol levels were predictive of a greater WR after BS. In our study, BS was highly effective in promoting weight reduction, with more than 75% of subjects achieving ≥30% TWL. The evidence regarding baseline cortisol as a marker of surgical response is contradictory. Consistent with our findings, other studies have also failed to identify differences in serum morning cortisol values between patients with optimal BS outcomes and those without [17]. In contrast, Bando et al. [18] showed that baseline cortisol < 10 μg/dL identified men who achieved greater weight loss after SG. In that study, the mean morning serum cortisol in men was lower than it was in our study (8.2 +/− 3.3 μg/dL vs. 13.1 [10.4–16.7 μg/dL]) and they had chosen TWL ≥ 25% at 1 year as the response; thus, the samples and endpoints are not comparable. Similarly, Janovik et al. [44] also reported an inverse correlation between morning serum cortisol at baseline and the degree of BMI reduction 6 months postoperative, but the difference at 12 months disappeared, losing clinical relevance. To our knowledge, there is only one paper evaluating cortisol levels after DST for predicting weight reduction after BS [17]. In that study, among 244 patients (79.1% female) who had undergone SG, a higher cortisol level after DST before surgery [0.6 (0.1–2.1) vs. 0.8 (0.7–1.1) μg/dL; p = 0.040] was associated to less probability of achieving %EWL ≥ 50% at 6 months after BS. In this study, a multivariate analysis is lacking, as is a longer follow-up. With respect to UFC, Muraca et al. [45] reported that a cortisol value above the normal range was associated with greater weight loss at 12 months after SG, as was younger age, lower HbA1c and lower impulsiveness. In this regard, we did not find an association between UFC and weight loss, even after a separate analysis by gender.

Regarding weight regain after BS, alarming figures have been reported [46]. King et al. [15] found that in SG patients, 5 years after reaching the nadir weight, subjects regained a median of 26.8% of the maximum weight lost. Several factors have been related to WR, including surgical technique, socioeconomic and behavioral factors, among others [47,48]. Despite controversial and heterogeneous results, the evidence suggests that WR is greater after SG than after RYGBG [49,50]. However, to our knowledge, there are no studies exploring cortisol levels in relation to weight rebound after BS. In our series, baseline serum morning cortisol was informative of WR (ρ 0.25, *p* < 0.01). A cut-off cortisol > 13 μg/dL showed the best performance regarding the detection of subjects at risk of regaining ≥ 10% of their lost weight at 2 years after BS. There are several studies using morning cortisol to assess stress and its consequences [51,52]. However, most studies have been conducted with salivary cortisol, because of the practicability and the measurement of the free form [13]. In this sense, although we did not determine CBG, we excluded subjects taking interferent drugs. There was a wide range of cortisol levels, but the median value among “regainers” was strikingly higher. On the one side, obese subjects usually show low levels of morning cortisol [22], but individuals with distinguishing features of metabolic syndrome generally display moderately elevated serum concentrations [44,53]. Our results could mean that those who regain weight have a maladapted HPA axis.

Among the mechanisms that could affect the relation between morning cortisol and WR, it is worth mentioning that various weight loss approaches impact cortisol levels. Caloric restriction from dieting can lead to increased cortisol levels [54], potentially contributing to weight regain due to elevated chronic psychological stress and cortisol production. However, the relationship between caloric restriction and cortisol levels is complex, as extremely reduced caloric intakes may elevate cortisol, while moderate restrictions may not necessarily do so; furthermore, changes in the cortisol/cortisone ratio have to be considered [55]. In this sense, significant weight loss after BS has been shown to reduce adipose tissue 11β-hydroxysteroid dehydrogenase type 1 expression, improving insulin sensitivity [56]. On the other hand, regular physical activity not only burns calories but also reduces stress and improves cortisol circadian rhythm, promoting a more favorable metabolic environment for weight loss [57,58]. Regarding weight-reducing drugs, GLP-1 is produced and has receptors in the central nervous system, which can stimulate the HPA axis [59,60]. However, there is limited evidence on the effect of GLP-1 analogs on cortisol levels in humans.

In addition, repetitive or chronic stress and other mental health conditions, as are frequent in some patients with SO, can dysregulate the HPA axis and alter cortisol levels, which could influence both the difficulty of losing weight and the likelihood of WR [61,62,63,64,65,66]. 

Cortisol can significantly impact WR through multiple mechanisms, such as promoting visceral fat accumulation, increasing appetite and reducing energy expenditure. Elevated cortisol levels can lead to insulin resistance and disrupt metabolic processes, creating a physiological environment that favors fat storage and weight gain. Additionally, chronic stress and cortisol dysregulation can drive emotional eating behaviors, further contributing to WR after significant weight loss.

Studies aimed at evaluating strategies that can promote hypercortisolism reduction in pwSO are warranted. In addition, these at-risk patients could benefit from early preventive strategies such us closer monitoring, targeted drugs (for instance, GLP-1, topiramate, etc.) [67,68] and a multidisciplinary approach to prevent WR [47,69]. All these approaches have proved to be successful in reversing WR and promoting further weight loss.

Our study has some limitations, such as the medium-size cohort and the short time of follow-up (24 months) to assess WR. Another limitation of our study was the conventional measurement of cortisol by immunoassay, since free cortisol measured by mass spectrometry has been shown to be more specific and accurate [70]. The main strength of the study is that common cortisol tests have been conducted within a well-characterized cohort of pwSO, in a real-life setting, with detailed follow-up after BS. Moreover, a multivariable analysis of cortisol results has been provided, taking into account comorbidities and confounder factors. We have contributed to increasing the scarce or absent knowledge about the prognostic value of cortisol tests with respect to weight loss. But, most important, this study is the first to explore pre-surgical cortisol tests and WR.

Our study dismissed the utility of baseline cortisol levels or dynamic tests as predictors of weight evolution after BS, except for morning serum cortisol, for which we provide a cutoff point that may be useful in clinical practice to comprehensively manage patients undergoing BS.

## 5. Conclusions

According to our results, none of the cortisol studies carried out before BS predicted weight loss, but an increased morning cortisol could warn of the tendency to regain weight. A pre-surgical morning serum cortisol > 13 μg/dL was predictive of a WR ≥ 10% at 2 years after BS and could be an easy tool to predict WR. Nevertheless, this finding requires confirmation using larger cohorts as well as a deeper investigation into the underlying mechanisms. Although our findings require validation, if confirmed, incorporating morning serum cortisol measurements into the holistic evaluation of pwSO could enable targeted stress-reduction interventions. Additionally, future research should explore the impact of stress-management strategies on cortisol levels and WR as well as compare cortisol variations across different weight loss approaches to better understand their influence on long-term outcomes.

## Figures and Tables

**Table 1:** Baseline characteristics of the patients by gender.

	Total, n: 142	Female, n: 101 (71.1%)	Male, n: 41 (28.9%)	
Age (y), mean ± SD	45.9 ± 9.2	44.8 ± 9.6	48.4 ± 7.6	0.04
BMI (kg/m^2^), median [IQR]	43.0 [40.4–47.5]	42.5 [40.1–47.1]	43.8 [40.7–48.2]	0.66
Waist (cm), median [IQR]	123 [115–131.5]	120 [110–125]	134 [128.75–143.5]	<0.01
Hip (cm), mean ± SD	135.5 ± 11.6	136.1 ± 1.0	133.6 ± 15.2	0.44
WHR, median [IQR]	0.9 [0.9–1.0]	0.9 [0.8–0.9]	1.02 [1.0–1.1]	<0.01
Excess weight (kg), median [IQR]	48.7 [39.9–63.2]	44.8 [33.4–58.5]	58.6 [44.2–73.3]	<0.01
Diabetes mellitus (n, %)	38 (26.8)	24 (23.8)	14 (34.1)	0.15
Hypertension (n, %)	39 (27.5)	21 (20.8)	18 (43.9)	<0.01
Dyslipidemia (n, %)	29 (20.4)	19 (18.8)	10 (24.4)	0.30
OSAS (n, %)	61 (43)	39 (38.6)	22 (55.0)	0.06
CPAP (n, %)	45 (31.7)	27 (26.7)	18 (45.0)	0.03
Smoking yes, n (%)	8 (5.6)	5 (5.0)	3 (7.3)	0.85
Serum morning cortisol (μg/dL), median [IQR]	12.0 [8.7–16.5]	11.6 [8.3–15.8]	13.1 [10.4–16.7]	0.13
DST (cortisol, μg/dL), median [IQR]	0.8 [0.6–1.0]	0.7 [0.6–1.0]	0.9 [0.5–1.3]	<0.01
Delta cortisol DST (μg/dL), median [IQR]	11.3 [8.1–15.5]	11.1 [7.8–15]	12.2 [9.7–15.5]	0.20
UFC (μg/24 h), median [IQR]	42.7 [24.2–67.2]	34.4 [20.5–58.5]	66.5 [46.9–125.0]	<0.01
LNSC (ng/mL), median [IQR]	0.75 [0.43–1.5]	0.6 [0.3–1.1]	0.9 [0.6–2.0]	0.12

Abbreviations: BMI: body mass index; WHR: waist-to-hip ratio; DST: 1 mg Dexamethasone suppression test; OSAS: obstructive sleep apnea syndrome; CPAP: continuous positive airway pressure; UFC: urinary free cortisol, LNSC: late-night salivary cortisol.

**Table 2:** Pre-surgical parameters according to total weight loss ≥ 30% at 12 months after bariatric surgery.

	TWL ≥ 30% n: 109 (76.8%)	TWL < 30% n: 33 (23.2%)	
Gender female, n (%)	76 (69.7%)	25 (75.8%)	0.33
Age (y), mean ± SD	45.2 ± 9.0	48.0 ± 9.8	0.12
Type of surgery, n (%)	RYGBP: 96 (88.1) SG: 13 (11.9)	RYGBP: 28 (84.8) SG: 5 (15.2)	0.41
Basal BMI (kg/m^2^), median [IQR]	43.93 [40.8–49.0]	41.3 [39.5–44.5]	<0.01
Excess weight (kg), median [IQR]	53 [40.3–65.7]	43.8 [39.1–51.0]	<0.01
WHR, median [IQR]	0.9 [0.9–1.0]	0.9 [0.8–1.0]	0.42
Diabetes mellitus, n (%)	26 (23.9)	12 (36.4)	0.16
OSAS, n (%)	45 (41.3)	16 (48.5)	0.38
CPAP, n (%)	34 (31.2)	11 (33.3)	0.84
Hypertension, n (%)	29 (26.6)	10 (30.3)	0.68
Serum morning cortisol (μg/dL), median [IQR]	12.0 [8.7–16.6]	12.5 [10.3–15.3]	0.83
DST (cortisol, μg/dL), median [IQR]	0.8 [0.6–1.0]	0.8 [0.7–1.2]	0.27
Delta cortisol (μg/dL), median [IQR]	11.1 [8.0–15.6]	12.0 [9.4–14.3]	0.33
UFC (μg/24 h), median [IQR]	45.6 [27.9–68.9]	26.6 [17.6–52.9]	0.46
LNSC (μg/dL), median [IQR]	0.8 [0.5–1.5]	0.6 [0.3–1.6]	0.62
ESR (mm/h), mean ± SD	43.3 ± 25.1	45.5 ± 25.3	0.65
IL-6 (pg/mL), mean ± SD	5.0 ± 3.2	4.0 ± 1.9	0.40
SHBG (nmol/L), mean ± SD	33.2 ± 20.0	34.1 ± 16.8	0.82
HOMA, mean ± SD	6.6 ± 6.5	5.7 ± 3.8	0.28

Abbreviations: RYGBP: Roux-en-Y gastric bypass; SG: sleeve gastrectomy; BMI: body mass index; OSAS: obstructive sleep apnea syndrome; CPAP: continuous positive airway pressure; DST: 1 mg Dexamethasone suppression test; UFC: urinary free cortisol, LNSC: late-night salivary cortisol; ESR: erythrocyte sedimentation rate; IL-6: interleukin-6; SHBG: steroid hormone binding globulin; HOMA: homeostatic model assessment.

**Table 3:** Weight regain ≥ 10% at 24 months after bariatric surgery.

	WR ≥ 10% n: 11 (7.8)	WR < 10% n: 131 (92.3)	
Gender female, n (%)	7 (63.6)	93 (71)	0.73
Age (y), mean ± SD	46 ± 9.9	45.9 ± 9.3	0.98
Type of surgery, n (%)	RYGBP 11 (100) SG 0	RYGBP 113 (86.3) SG 18 (13.7)	0.36
BMI (kg/m^2^), median [IQR]	42.78 [41.4–49.7]	43.0 [40.1–47.2]	0.88
Excess weight (kg), median [IQR]	55 [42–72.6]	48.7 [39.3–60.8]	0.52
WHR, median [IQR]	1.0 [0.9–1.1]	0.9 [0.9–1.1]	0.18
Diabetes mellitus, n (%)	4 (36.4)	33 (25.2)	0.49
OSAS, n (%)	4 (36.4)	52 (39.7)	0.70
CPAP use, n (%)	4 (36.4)	38 (29.0)	0.74
Hypertension, n (%)	2 (18.2)	35 (26.7)	0.47
Serum morning cortisol (μg/dL), median [IQR]	17.8 [13.1–18.5]	12.0 [8.8–15.8]	<0.01
DST (cortisol μg/dL), median [IQR]	0.8 [0.6–1.0]	0.8 [0.6–1.0]	0.43
Delta cortisol (μg/dL), median [IQR]	16.89 [12.24–17.5]	11.15 [7.97–14.7]	<0.01
UFC (μg/24 h), median [IQR]	41.4 [17.6–105.6]	45.0 [24.5–67.2]	0.92
LNSC (μg/dL), median [IQR]	0.7 [0.1–1.8]	0.8 [0.5–1.5]	0.67
ESR (mm/h), mean ± SD	42 ± 29.1	44.4 ± 24.8	0.77
SHBG (nmol/L), mean ± SD	26.0 ± 11.0	33.9 ± 20.0	0.24
HOMA, mean ± SD	7.8 ± 6.4	6.4 ± 6.0	0.45
IL-6 (pg/mL), mean ± SD	5.1 ± 0.1	4.7 ± 3.0	0.89

Abbreviations: BMI: body mass index; OSAS: obstructive sleep apnea syndrome; CPAP: continuous positive airway pressure; DST: 1 mg Dexamethasone suppression test; UFC: urinary free cortisol, LNSC: late-night salivary cortisol; ESR: erythrocyte sedimentation rate; IL-6: interleukin-6; SHBG: steroid hormone binding globulin; HOMA: homeostatic model assessment.

**Table 4:** Logistic regression analysis for weight regain ≥ 10% at 2 years after BS.

Variable	OR	95% CI for OR	*p* Value
Serum morning cortisol	1.216	1.069–1.384	0.003
Gender	2.842	0.322–25.097	0.347
BMI	0.733	0.480–1.119	0.150
EW	1.120	0.966–1.299	0.134
Type of BS	1.477	0.283–7.703	0.643
HOMA	0.993	0.887–1.111	0.899

Abbreviations: BMI: body mass index; EW: excess weight; BS: bariatric surgery.

## Data Availability

The dataset supporting this study is available upon request from the authors.
